# An observational cohort study on the effects of extended postoperative antibiotic prophylaxis on surgical-site infections in low- and middle-income countries

**DOI:** 10.1093/bjs/znad438

**Published:** 2024-01-10

**Authors:** Clean Cut Investigators Group, Maia R. Nofal, Alex Y. Zhuang, Natnael Gebeyehu, Nichole Starr, Sara Taye Haile, Habtamu Woldeamanuel, Assefa Tesfaye, Senait Bitew Alemu, Abebe Bekele, Tihitena Negussie Mammo, Thomas G. Weiser, Abdi Amin Abdukadir, Belay Mellese Abebe, Ananya K. Admasu, Tibebu Abebe Alito, Reshma Ambulkar, Sedera Arimino, Muhudin Arusi, Nardos Aynalem, Varnica Bajaj, Tilahun Selfago Delelo, Motuma Gutu, Feleke Habte, Gezahegn Assefa Hurrisa, Aditya Kunte, Karoline Rocabado, Matiyas Asrat Shiferaw, Constance Harrell-Shreckengost, Agazi Tiruneh, Roberto Zamorano, Milena Abreha, Constanza Aguilera, Bella Lima, Hillena Kebede

## Abstract

**Background:**

Worldwide, approximately one in six inpatient antibiotic prescriptions are for surgical-infection prophylaxis, including postoperative prophylaxis. The WHO recommends against prolonged postoperative antibiotics to prevent surgical-site infection. However, in many low- and middle-income countries, postoperative antibiotic prophylaxis is common due to perceptions that it protects against surgical-site infection and data informing recommendations against antibiotic administration are largely derived from high-income countries. The aim of this study was to describe postoperative antibiotic-prescribing patterns and related surgical-site infection rates in hospitals in low- and middle-income countries.

**Methods:**

Patients from 19 hospitals in Ethiopia, Madagascar, India, and Bolivia with wound class I and II operations were included. Data on antibiotic administration, indication, surgical-site infection, length of hospital stay, and adherence to perioperative infection-prevention standards were collected by trained personnel. The association between postoperative antibiotic prophylaxis for greater than or equal to 24 h and surgical-site infection was analysed via modified robust Poisson regression, controlling for patient and procedural factors and degree of adherence to perioperative infection-prevention practices.

**Results:**

Of 8714 patients, 92.9% received antibiotics for prophylaxis after surgery and 27.7% received antibiotics for greater than or equal to 24 h. Patients receiving postoperative prophylaxis for greater than or equal to 24 h did not have lower surgical-site infection rates (Relative risk 1.09 (95% c.i. 0.89 to 1.33); *P* = 0.399), but the length of hospital stay was 1.4 days longer (*P* < 0.001).

**Conclusion:**

Prolonged postoperative antibiotics did not reduce surgical-site infection, but pervasive use was associated with a longer length of hospital stay, in resource-limited healthcare systems. With the growing threat of antimicrobial resistance, surgical initiatives to implement antimicrobial stewardship programmes in low- and middle-income countries are critical.

## Teaser

This cohort study evaluated whether postoperative antibiotic prophylaxis decreased the risk of surgical-site infections in 19 hospitals in low- and middle-income countries, where postoperative prophylaxis is common. Administering postoperative prophylaxis for greater than or equal to 24 h was not associated with a lower risk of surgical-site infection.

## Introduction

Since the discovery of penicillin in the early 1920s revolutionized the practice of medicine and surgery, antimicrobial resistance has risen exponentially. Resistance patterns increasingly continue to threaten healthcare systems and the ability to deliver effective patient care^1^. In 2019, antimicrobial resistance was estimated to be associated with nearly 5 million deaths^2^. If antimicrobial resistance continues on its current trajectory, it is estimated that antimicrobial resistance will threaten 10 million lives and cost 92 trillion euros per year by 2050^3^.

The highest burden of antimicrobial resistance worldwide is thought to be in sub-Saharan Africa^2^, although more than 40% of the countries in the continent of Africa do not have any data available regarding resistance patterns^4^. In countries with published data, staggeringly high levels of resistance to commonly used antibiotics have been found^5,6^. Surgical antibiotic prophylaxis makes up approximately one in six antibiotic prescriptions worldwide, including postoperative antibiotics for the prevention of surgical-site infection (SSI)^7^. The WHO recommends against the use of postoperative antibiotic prophylaxis due to strong evidence that it has no benefit in preventing SSI^8,9^. However, postoperative prophylaxis remains common in low- and middle-income countries (LMICs) due to perceptions that these recommendations are largely derived from high-income countries and thus do not apply to LMIC hospitals, where sterility practices are variable and SSI rates are much higher^10–13^. The lack of contextually appropriate data remains a barrier to antibiotic-stewardship efforts in LMICs, particularly in sub-Saharan Africa. Further investigation is needed to understand current practices and identify opportunities for antimicrobial stewardship. The aim of this study was to describe postoperative antibiotic-prescribing practices and assess the impact of these practices on SSI in surgical patients with wound class I and II operations at LMIC hospitals.

## Methods

### Study design and inclusion criteria

This was an observational cohort study involving a secondary analysis of data collected as part of the Clean Cut programme, a surgical quality-improvement programme run by Lifebox aimed at reducing SSI^14^. Data from 19 hospitals were collected from 2019 to 2022, including 16 hospitals in Ethiopia, 1 hospital in Madagascar, 1 hospital in India, and 1 hospital in Bolivia, where the Clean Cut programme was ongoing. At the start of the quality-improvement programme, operating rooms were identified to enrol patients; enrolment occurred at any time of day or night and included patients of any age undergoing any type of operation. The majority of hospitals were referral or tertiary centres, with the exception of one hospital in Ethiopia and one hospital in Madagascar. The results of the initial implementation of the Clean Cut programme are described elsewhere^15^. Because this was a secondary analysis of a quality-improvement programme, ethical approval was not specifically obtained for this study. The study focused on patients who underwent clean (wound class I) or clean-contaminated (wound class II) operations, because these patients typically do not require postoperative antibiotics for the treatment of known active infections, abscesses, or gross wound contamination. All patients from the 19 hospitals who underwent clean or clean-contaminated operations and were admitted to a ward after surgery were included. Patients who were documented in their chart as having an infection within the first 48 h after surgery were excluded to ensure that all included patients were being given antibiotics for prophylaxis rather than for clinical suspicion of infection. Prolonged postoperative antibiotic prophylaxis was defined as greater than or equal to 24 h of antibiotic prophylaxis after surgery. The primary outcome was 30-day SSI rates, as defined by the US Centers for Disease Control and Prevention (‘CDC’) and operationalized as pus draining from the wound, a previously closed wound opened intentionally due to infection, or an abscess, organ space infection, or deep SSI on imaging^16^. Secondary outcomes included indication and choice of antibiotic and length of hospital stay.

### Data collection

Trained personnel collected clinical data in the operating room, such as medical co-morbidities, case urgency, wound class, and adherence to six key standards of infection prevention: hand and skin antisepsis; sterile field preparation; instrument sterility; antibiotic administration; gauze counting; and use of the WHO Surgical Safety Checklist. Surgical teams were only considered compliant with each standard if they completed all observable behaviours associated with that standard (*Table 1*). Data on postoperative antibiotic administration were collected by trained nurses daily through review of inpatient charts, focusing on whether a patient was prescribed antibiotics and for what indication. The presence of other infections, such as urinary tract infections, endometritis, and pneumonia, was also captured through chart review. SSI rates were captured during admission through wound inspection by trained data collectors, chart documentation by treating physicians, and via telephone on postoperative day 30, when data collectors asked targeted questions about the surgical incision, including erythema around the wound, wound drainage, and wound dehiscence.

**Table 1:** Observable behaviours required to meet infection-prevention standards

Infection-prevention standard	Components required for compliance
Hand and skin antisepsis	Surgical hand scrub with appropriate soap or alcohol-based hand rub
	Surgical-site skin appropriately prepared
	New gloves worn by surgeon
Sterile field preparation	Sterile indicator was present inside gown and drape pack
	Sterile indicator changed colour indicating appropriate sterilizing procedures
	Gowns and drapes were dry and without holes
Instrument sterility	Sterility indicator was present inside instrument tray
	Sterility indicator changed colour indicating appropriate sterilizing procedures
	Instrument tray was dry
Antibiotic administration	Antibiotics were given before surgery
	Antibiotics were given within 60 min of incision or in the operating room
Gauze counting	Gauze was counted before the operation
	Gauze was counted after the operation
Use of the WHO Surgical Safety Checklist	A sign in was completed aloud before induction of anaesthesia
	A timeout was performed before incision
	A sign out was completed aloud at the end of the procedure in the operating room

### Statistical analysis

Patient demographic and procedure characteristics were compared between those who received prolonged postoperative antibiotic prophylaxis and those who did not. The relative risk of SSI for patients who received postoperative prophylactic antibiotics was assessed using modified robust Poisson regression, controlling for demographic and clinical factors, such as age, sex, wound class, case urgency, surgical specialty, and degree of adherence to perioperative infection-prevention practices. The degree of adherence to perioperative infection-prevention practices was scored (out of six total practices) by the number of areas of infection-prevention standards met by the surgical team (*Table 1*). High adherence was defined as meeting at least three of six standards, as adherence to three of six perioperative infection-prevention practices was previously demonstrated to be associated with lower rates of SSI in low-resource settings^15^. Due to loss to follow-up when 30-day telephone interviews were conducted, sensitivity analyses were conducted using best- and worst-case scenarios. In the best-case scenario, all patients lost to follow-up were assumed not to have an infection and included in the analysis; in the worst case scenario, patients lost to follow-up were assumed to have an infection and included in the analysis. Finally, differences in the length of hospital stay between patients who received prolonged postoperative antibiotics and those who did not were analysed using Student’s *t* test. The relationship between length of hospital stay and days of postoperative antibiotic prophylaxis was analysed via linear regression with robust standard errors controlling for the same demographic and clinical characteristics. All analyses were conducted using Stata version 16.1/standard edition (College Station, TX, USA).

## Results

**Fig. 1:**
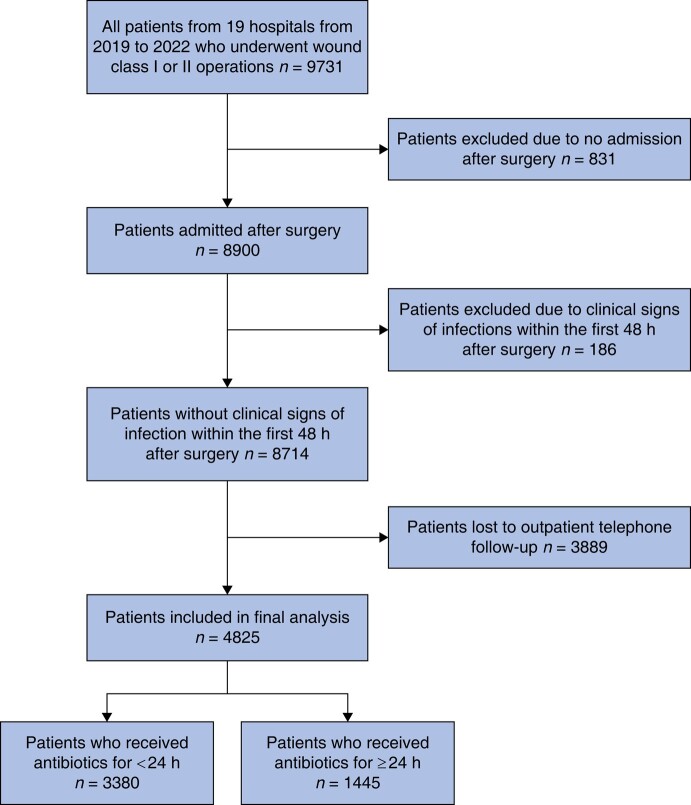
Inclusion criteria for the primary outcome

Some 9731 patients from 19 hospitals who underwent clean or clean-contaminated operations were identified. Of those, 8900 patients (91.5%) were admitted after surgery and were included in the analysis. A total of 186 patients (2.1%) who were identified as having clinical signs of infection within the first 48 h after surgery were excluded, leaving 8714 patients (89.5%) who met the inclusion criteria, of whom 8101 (93.0%) were from Ethiopia. Of these patients meeting the inclusion criteria, 6297 patients (72.2%) received antibiotics for less than 24 h, whereas 2417 patients (27.7%) received antibiotics for greater than or equal to 24 h, and 3889 patients (44.6%) could not be reached for the 30-day telephone follow-up. Ultimately, 4825 patients were included in the analysis of the primary outcome (*Fig. 1*).

Patient characteristics were roughly comparable between patients who received prolonged (greater than or equal to 24 h) postoperative antibiotics for prophylaxis and those who did not, although there were more clean cases and more emergency operations in the former group and more risk factors in the latter group (*Table 2*). Caesarean sections corresponded to the largest proportion of operations. A detailed breakdown of the case mix is included in *Table S1*. Adherence to infection-prevention practices was significantly higher in the group that received greater than 24 h of postoperative prophylaxis, with an average score of 3.5 of 6 compared with 2.9 of 6 (*Table 2*).

**Table 2:** Patient characteristics

	Length of postoperative antibiotic prophylaxis <24 h (*n* = 6297)	Length of postoperative antibiotic prophylaxis ≥24 h (*n* = 2417)
**Age (years), mean(s.d.)**	28.4(10.4)	27.5(12.2)
**Male**	362 (5.7)	317 (13.1)
**Hypertension**	272 (4.3)	78 (3.2)
**Diabetes**	77 (1.2)	27 (1.1)
**Wound class**		
Clean	2760 (43.8)	1553 (64.3)
Clean-contaminated	3537 (56.2)	864 (35.7)
**Procedure type**		
General surgery	428 (6.8)	200 (8.3)
Obstetric	5080 (80.7)	1808 (74.8)
Sub-specialty surgery	192 (3.0)	309 (12.8)
Gynaecology	597 (9.5)	100 (4.1)
**Case urgency**		
Elective	2226 (35.4)	626 (25.9)
Emergency	4071 (64.6)	1791 (74.1)
**Adherence to perioperative infection-prevention standards**		
Patients with high compliance (≥3 of 6 areas)	3942 (62.3)	1733 (71.7)
Number of areas of compliance with infection prevention out of six, mean(s.d.)	2.9(1.4)	3.5(1.5)

Values are *n* (%) unless otherwise indicated.

**Fig. 2:**
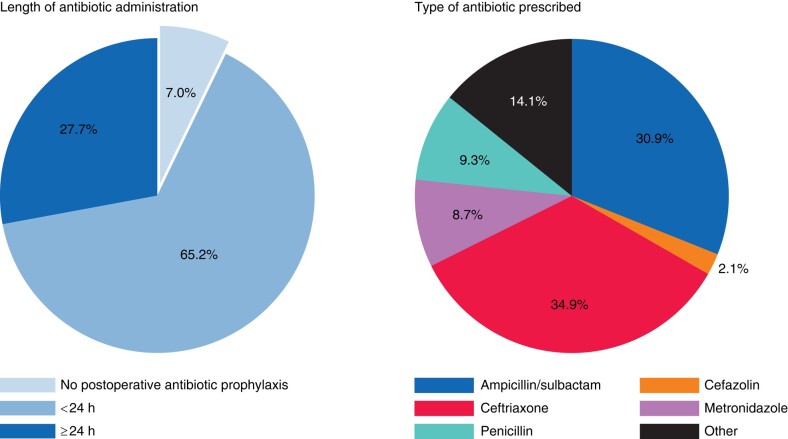
Postoperative antibiotic prophylaxis prescribing patterns

Nearly all patients received antibiotics after surgery for prophylaxis; only 7% (614 of 8714) did not receive any prophylaxis after surgery. Typically a single medication was given for prophylaxis; ceftriaxone was the most frequently prescribed, followed by ampicillin/sulbactam (*Fig. 2*). Indications for antibiotics prescribes for reasons apart from prophylaxis are shown in *Table S4*.

The SSI rate among patients who had less than 24 h of prophylaxis was 9.8% (331 of 3380) compared with 11.9% (172 of 1445) among patients who had prolonged courses of antibiotics. After modified Poisson regression, controlling for age, sex, wound class, case urgency, surgical specialty, and degree of adherence to perioperative infection-prevention practices, there was no significant difference in the risk of SSI between patients who received prolonged postoperative prophylactic antibiotics and those who did not (Relative risk (RR) 1.08 (95% c.i. 0.88 to 1.31); *P* = 0.468) (*Table 3*). However, high adherence to perioperative infection-prevention practices was associated with a significantly lower risk of SSI (RR 0.73 (95% c.i. 0.61 to 2.03); *P* = 0.001). Sub-specialty surgery, which included primarily head and neck and orthopaedic as well as vascular, neurological, and urological surgery, was associated with significantly higher rates of SSI compared with other procedure types.

**Table 3:** Relative risk of SSI after greater than or equal to 24 h of postoperative antibiotic prophylaxis, controlling for clinical and procedural characteristics; *n* = 4825

	RR (95% c.i.)	*P*
**Length of postoperative antibiotic prophylaxis**		
<24 h (reference)	1	–
≥24 h	1.09 (0.89,1.33)	0.399
**Sex**		
Female (reference)	1	–
Male	1.16 (0.81,1.67)	0.420
**Age (years)**		
<20 (reference)	1	–
20–29	1.58 (1.11,2.24)	0.011
30–39	1.56 (1.09,2.25)	0.015
≥40	1.46 (0.97,2.19)	0.068
**Hypertension**	1.18 (0.80,1.75)	0.395
**Diabetes**	1.59 (0.92,2.74)	0.096
**Procedure type**		
General surgery (reference)	1	–
Obstetric	0.79 (0.53,1.20)	0.271
Sub-specialty surgery	1.82 (1.26,2.64)	0.002
Gynaecology	0.93 (0.55,1.56)	0.784
**Adherence to perioperative infection prevention**		
Low adherence (<3 of 6 standards met) (reference)	1	–
High adherence (≥3 of 6 standards met)	0.74 (0.61,0.89)	0.001
**Wound class**		
Clean (reference)	1	–
Clean-contaminated	1.21 (1.01,1.45)	0.036
**Case urgency**		
Elective (reference)	1	–
Urgent or emergency	1.37 (1.10,1.70)	0.004

RR, Relative risk.

A large number of patients (44.6%) were lost to follow-up; the characteristics of the patients who were lost to follow-up and those who were not were similar, although a slightly higher percentage of obstetric cases were lost to follow-up (*Table S2*). To account for loss to follow-up, sensitivity analyses using best- and worst-case scenarios were conducted. In the best-case scenario, where patients were assumed to have no SSI, prolonged antibiotics did not affect the risk of SSI (RR 1.17 (95% c.i. 0.96 to 1.43); *P* = 0.114). Similarly, prolonged antibiotic prophylaxis did not affect the risk of SSI in the worst-case scenario, where patients lost to follow-up were all assumed to have an SSI (RR 0.95 (95% c.i. 0.89 to 1.02); *P* = 0.178). Details of these sensitivity analyses are included in *Table S3*.

Finally, the mean length of hospital stay was higher in the group that received prolonged antibiotic prophylaxis compared with the group that did not (3.67 *versus* 2.30 days respectively; *P* < 0.001). There was a positive relationship between days of antibiotic prophylaxis and length of hospital stay, where 51% of variation in length of hospital stay could be explained by the length of antibiotic prophylaxis after controlling for other clinical factors (coefficient 0.71, R-squared 0.51 (95% c.i. 0.62 to 0.79); *P* < 0.001).

## Discussion

In this large, observational cohort study, prolonged postoperative antibiotic prophylaxis did not reduce 30-day SSI rates, but was associated with a significantly longer length of hospital stay. Instead, lower SSI rates were driven primarily by improved adherence to perioperative infection-prevention practices; notably, adherence to infection-prevention practices was actually higher in the group that received prolonged postoperative prophylaxis. Longer hospitalizations may be influenced by extended prophylactic antibiotic administration. Because this has implications for bed utilization and costs to both health systems and patients who frequently bear additional out-of-pocket costs, further investigation is needed to understand if improving stewardship practices can also reduce the length of hospital stay.

Because recommendations against postoperative antibiotic prophylaxis are largely based on data from high-income countries, surgeons working in LMICs may question their relevance. For example, the Ethiopian Food, Medicine and Healthcare Administration and Control Authority partnered with the US Agency for International Development (‘USAID’) Global Health Supply Chain Program to develop standard treatment guidelines for antibiotic use in the prevention and treatment of SSI, but adherence to antibiotic choice, length of treatment, and therapy de-escalation recommendations remains low^17–19^. Mistrust of guidelines is understandable when the local context differs with regard to sterility practices and infection-prevention systems. However, the present study focused on context-relevant settings and further controls for adherence to infection-prevention practices, which can vary widely among hospitals. Infection-prevention practices were more relevant in driving SSI rates than postoperative antibiotic prophylaxis. Further research on the implementation and impact of these infection-prevention practices are underway. In the present cohort, postoperative antibiotic prophylaxis was common and, in nearly one-third of patients, was continued for 24 h or longer after surgery. This practice of extending antibiotic prophylaxis after surgery contributes to worsening antibiotic resistance^8^, a problem that disproportionately affects LMICs, particularly sub-Saharan African countries^5,6^. In LMIC hospitals, where SSI rates are high, prolonging prophylaxis after surgery is often considered part of common practice to prevent surgical infections, calling into question the relevance of international recommendations. The results of the present study reinforce the applicability of international recommendations to LMIC contexts and emphasize a need for a shift in practice, focused on strengthening systems of infection prevention in operating rooms rather than extending postoperative antibiotics.

One limitation of the present study is the loss to follow-up for the primary outcome. The group with complete follow-up had fewer women, fewer obstetric patients, and fewer emergency operations compared with the group lost to follow-up. Unfortunately, patient follow-up is a persistent challenge in the settings where the data were collected. Many patients travel long distances for medical care and may not have consistent or reliable access to telephone and internet connections. To account for attrition bias, sensitivity analyses assumed all missing patients either had or did not have an SSI, an approach that allowed the authors to bias their results in favour of an SSI reduction and against an SSI reduction in turn. In both analyses, no reduction in SSI was found in the group receiving prolonged antibiotic prophylaxis.

The present study is also limited by its approach as a secondary analysis of an existing programme. The authors pursued this secondary analysis driven by their study team’s observations around prescribing practices while reviewing data from the Clean Cut programme. However, this limited the inclusion criteria to those patients whose data had already been collected, which may have introduced selection bias. Additionally, the design of the study meant that the clinical reasons why some patients received extended antibiotics and others did not could not be captured. The authors intend to pursue further studies with a prospective approach. Additionally, while a variety of different hospitals in four countries were included, the conclusions may not be generalizable to all LMIC settings. The data were mostly from hospitals in Ethiopia and may best reflect practice patterns in this setting, as compared with Bolivia, India, and Madagascar, where only a single hospital was included for each of these countries. The majority of hospitals were referral hospitals, which may differ in terms of antibiotic-prescribing patterns and infection-prevention practices. Furthermore, the patient population had a significant number of women undergoing caesarean sections, reflecting how commonplace this procedure is in the settings that were studied. There were relatively few patients who underwent operations requiring hardware or implants. The limited case mix may limit the generalizability of the present study.

While the present study addresses concerns around the applicability of international recommendations to LMIC contexts, this is only one component of antibiotic stewardship. Further barriers include limited data on local resistance patterns, poor education on antimicrobial resistance, and a failure of national guidelines to drive evidence-based practice^20^. Data on antimicrobial resistance patterns are lacking in LMICs, particularly in sub-Saharan Africa. When resistance patterns are available, high levels of resistance to commonly used antibiotics are found^6^. This is reflected in the present study as well, where the most frequently prescribed antibiotics for the patient cohort, ceftriaxone and ampicillin, have reported resistance rates that are as high as 69% and 60% respectively in East Africa^5^. Most hospitals included in the analysis did not have culture capabilities. As a result, the majority of hospitals included in the present study did not have a local antibiogram or a standardized protocol for choosing which antibiotic to prescribe. Instead, antibiotic availability is limited in many LMICs, which may have influenced the choice of antibiotic for prophylaxis^21^. Additionally, qualitative studies have shown that many clinicians in these settings feel that broad-spectrum antibiotics pose a low risk for worsening antimicrobial resistance, especially in systems where patients can choose to take antibiotics that are freely available at pharmacies without a prescription^20^. Overprescribing may also be influenced by requests by patients, who may associate antibiotics with proper treatment and request prescriptions, even when not indicated^22^. While reasons for antibiotic misuse and overuse in LMICs have been studied, further research is needed on the drivers of these behaviours with regards to surgical patients. For example, in the cohort of the present study, surprisingly, patients with clean wounds had higher rates of prolonged prophylaxis. These hospitals had no routine surveillance of infection-prevention practices before the start of the Clean Cut programme. While clinicians may observe high infection rates in their patients, identifying specific breakdowns in the infection-prevention system would be challenging on a patient basis. Anecdotally, clinician have described additional concerns that may factor into the decision to extend prophylaxis as an additional precaution, including hospital ward hygiene, the ability to maintain a clean surgical wound at home, and access to postoperative care upon discharge. These areas require further research to better understand the effect of these factors on prescribing behaviours.

Finally, many LMICs, particularly sub-Saharan African countries, may not have robust antimicrobial stewardship guidelines; when guidelines are available, they often fail to account for local disease burden or drug availability, so adherence is poor^23^. While changing practices may be challenging, improvements in stewardship efforts are possible. For example, a single-centre study in Kenya found that a quality-improvement programme that applied local standard treatment guidelines to antibiotic-prescribing practices reduced unnecessary postoperative antibiotic prescriptions^24^. While not specifically targeting surgical patients, a number of similar programmes have been successful in antibiotic-stewardship efforts in African countries^25^. Concerted efforts to address antibiotic stewardship barriers are urgently needed, especially among the surgical community, who prescribe a significant proportion of antibiotics globally^7^.

## Collaborators

**Clean Cut Investigators Group**

Writing Group

Maia R. Nofal (Boston Medical Center, Department of Surgery, Boston University Chobanian & Avedisian School of Medicine, Boston, Massachusetts, USA; Stanford University, Department of Surgery, Palo Alto, California, USA; Fogarty International Center, Global Health Equity Scholars Program (D43TW010540), Washington DC, USA; Lifebox Foundation, Addis Ababa, Ethiopia); Alex Y. Zhuang (Boston University Chobanian & Avedisian School of Medicine, Boston, Massachusetts, USA; Lifebox Foundation, Addis Ababa, Ethiopia; Fogarty International Center, Harvard-BU-Northwestern-UNM Consortium (D43TW010543), Washington DC, USA; University of Global Health Equity, Kigali, Rwanda); Natnael Gebeyehu (Lifebox Foundation, Addis Ababa, Ethiopia; Addis Ababa University, Department of Surgery, Ethiopia); Nichole Starr (Lifebox Foundation, Addis Ababa, Ethiopia; University of California San Francisco, Department of Surgery, San Francisco, California USA); Sara Taye Haile (Lifebox Foundation, Addis Ababa, Ethiopia); Habtamu Woldeamanuel (Lifebox Foundation, Addis Ababa, Ethiopia); Assefa Tesfaye (Lifebox Foundation, Addis Ababa, Ethiopia; St. Peter’s Specialized Hospital, Addis Ababa, Ethiopia); Senait Bitew Alemu (Lifebox Foundation, Addis Ababa, Ethiopia); Abebe Bekele (University of Global Health Equity, Kigali, Rwanda); Tihitena Negussie Mammo (Lifebox Foundation, Addis Ababa, Ethiopia; Addis Ababa University Department of Surgery, Ethiopia); Thomas G. Weiser (Stanford University, Department of Surgery, Palo Alto, California, USA; Lifebox Foundation, Addis Ababa, Ethiopia).

Other Collaborators

Abdi Amin Abdukadir (Haramaya University, College of Health and Medical Sciences,Hiwot Fana Comprehensive Specialized Hospital, Department of Surgery, Harar, Ethiopia); Belay Mellese Abebe (Hawassa University Comprehensive Specialized Hospital, Department of Surgery, Hawassa, Ethiopia); Ananya K. Admasu (University of Gondar, Department of Orthopedic Surgery, Gondar, Ethiopia); Tibebu Abebe Alito (Yirgalem General Hospital, Department of Obstetrics and Gynecology, Yirgalem, Ethiopia); Reshma Ambulkar (Tata Memorial Centre, Homi Bhabha National Institute, Department of Anesthesia, Mumbai, Maharashtra, India); Sedera Arimino (Lifebox Foundation, Addis Ababa, Ethiopia); Muhudin Arusi (Werabe Comprehensive Specialized Hospital, Department of Obstetrics and Gynecology, Werabe, Ethiopia); Nardos Aynalem (ALERT Comprehensive Hospital, Department of Obstetrics and Gynecology, Addis Ababa, Ethiopia); Varnica Bajaj (University of Nebraska Medical Center, Department of Surgery, Omaha, Nebraska, USA); Tilahun Selfago Delelo (Adare General Hospital, Department of Obstetrics and Gynecology, Hawassa, Ethiopia); Motuma Gutu (Ambo University, Department of Obstetrics and Gynecology, Ambo, Ethiopia); Feleke Habte (Wolkite University Specialized Teaching Hospital, Department of Obstetrics and Gynecology, Wolkite, Ethiopia); Gezahegn Assefa Hurrisa (Yekatit 12 Hospital Medical College, Department of Surgery, Addis Ababa, Ethiopia); Aditya Kunte (Tata Memorial Centre, Homi Bhabha National Institute, Department of Surgery, Mumbai, Maharashtra, India); Karoline Rocabado (Oncology Institute of Eastern Bolivia, Santa Cruz, Bolivia); Matiyas Asrat Shiferaw (St. Paul’s Hospital Millennium Medical College, Department of Obstetrics and Gynecology, Addis Ababa, Ethiopia); Constance Harrell-Shreckengost (Emory University, Department of Surgery, Atlanta, Georgia, USA); Agazi Tiruneh (Zewditu Memorial Hospital, Department of Surgery, Addis Ababa, Ethiopia); Roberto Zamorano (Hospital San José de Osorno, Department of Anesthesia, Los Lagos, Chile); Milena Abreha (Lifebox Foundation, Addis Ababa, Ethiopia); Constanza Aguilera (Lifebox Foundation, Addis Ababa, Ethiopia); Bella Lima (Lifebox Foundation, Addis Ababa, Ethiopia); Hillena Kebede (Lifebox Foundation, Addis Ababa, Ethiopia).

## Supplementary Material

znad438_Supplementary_Data

## Data Availability

Deidentified data will be made available on reasonable request.
