# Supplementary material for: An observational cohort study on the effects of extended postoperative antibiotic prophylaxis on surgical-site infections in low- and middle-income countries
Source: Br J Surg. 2024 Jan 10;111(1):znad438. doi: 10.1093/bjs/znad438 (PMC10782210; doi:10.1093/bjs/znad438)
Supplement: znad438_Supplementary_Data [file znad438_supplementary_data.docx]

Effects of extended postoperative antibiotic prophylaxis on surgical site infections in low- and middle-income countries: a cohort study of nineteen hospitals

Clean Cut Investigators Group

**Corresponding author.** Maia R. Nofal, MD MPH, Surgery Education Office, Boston Medical Center, Boston University Chobanian & Avedisian School of Medicine, 85 E. Concord Street, Third Floor, Boston, Massachusetts, 02118, USA (e-mail: Maia.Nofal@bmc.org)

ORCID ID: 0000-0003-2532-4514, Twitter: @MaiaNofal

**Supplementary Materials - Index**

| **Supplementary Appendixes** |  |
| --- | --- |
| Collaborators | *pag. 2* |
| **Supplementary Figures and Tables** |  |
| Supplementary table 1 | *pag. 3* |
| Supplementary table 2 | *pag. 3* |
| Supplementary table 3 | *pag. 4* |
| Supplementary table 4 | *pag. 5* |
|  |  |
|  |  |

**Supplementary Appendixes**

The following are a complete list of authors, collaborators, and their associated roles and affilliations:

Writing group:

Maia R. Nofal, MD MPH^1,2,3,4^, Alex Y. Zhuang, MS^1,4,5,6^, Natnael Gebeyehu, MD^4,7^, Nichole Starr, MD MPH^3,4,8^, Sara Taye Haile, MPH^4^, Habtamu Woldeamanuel^4^, Assefa Tesfaye, MD MPH^4,9^, Senait Bitew Alemu BSc, MPH^4^, Abebe Bekele MD^6^, Tihitena Negussie Mammo, MD^4,7^, Thomas G. Weiser, MD MPH^2,4^

1. Boston Medical Center, Department of Surgery, Boston University Chobanian & Avedisian School of Medicine, Boston, Massachusetts, USA

2. Stanford University, Department of Surgery, Palo Alto, California, USA

3. Fogarty International Center, Global Health Equity Scholars Program (D43TW010540), Washington DC, USA

4. Lifebox Foundation, Addis Ababa, Ethiopia

5. Fogarty International Center, Harvard-BU-Northwestern-UNM Consortium (D43TW010543), Washington DC, USA

6. University of Global Health Equity, Kigali, Rwanda

7. Addis Ababa University, Department of Surgery, Ethiopia

8. University of California San Francisco, Department of Surgery San Francisco, California USA

9. St. Peter’s Specialized Hospital, Addis Ababa, Ethiopia

Clean Cut Clinical Collaborators:

Abdi Amin Abdukadir, MD^1^, Belay Mellese Abebe MD^2^, Ananya K. Admasu, MD^3^, Tibebu Abebe Alito, MD^4^, Reshma Ambulkar MD^5,6^, Sedera Arimino MD^6^, Muhudin Arusi MD^7^, Nardos Aynalem MD^8^, Varnica Bajaj, MD^9^, Tilahun Selfago Delelo^10^, Motuma Gutu MD^11^, Feleke Habte, MD MPH^12^, Gezahegn Assefa Hurrisa^13^, Aditya Kunte, MD^5^, Karoline Rocabado, MD^14^, Matiyas Asrat Shiferaw, MD^6,15^, Constance Harrell-Shreckengost, MD, PhD^16^, Agazi Tiruneh, MD^17^, Roberto Zamorano MD^6,^

1. Haramaya University, College of Health and Medical Sciences,Hiwot Fana Comprehensive Specialized Hospital, Department of Surgery, Harar, Ethiopia

2. Hawassa University Comprehensive Specialized Hospital, Department of Surgery, Hawassa, Ethiopia

3. University of Gondar, Department of Orthopedic Surgery, Gondar, Ethiopia

4. Yirgalem General Hospital, Department of Obstetrics and Gynecology, Yirgalem, Ethiopia

5. Tata Memorial Centre, Homi Bhabha National Institute, Mumbai, Maharashtra, India

6. Lifebox Foundation, Addis Ababa, Ethiopia

7. Werabe Comprehensive Specialized Hospital, Department of Obstetrics and Gynecology, Werabe, Ethiopia

8. ALERT Comprehensive Hospital, Department of Obstetrics and Gynecology, Addis Ababa, Ethiopia

9. University of Nebraska Medical Center, Department of Surgery, Omaha, Nebraska, USA

10. Adare General Hospital, Department of Obstetrics and Gynecology, Hawassa, Ethiopia

11. Ambo University, Department of Obstetrics and Gynecology, Ambo, Ethiopia

12. Wolkite University Specialized Teaching Hospital, Department of Obstetrics and Gynecology, Wolkite, Ethiopia

13. Yekatit 12 Hospital Medical College, Department of Surgery, Addis Ababa, Ethiopia

14. Oncology Institute of Eastern Bolivia, Santa Cruz, Bolivia

15. St. Paul’s Hospital Millennium Medical College, Department of Obstetrics and Gynecology, Addis Ababa, Ethiopia

16. Emory University, Department of Surgery, Atlanta, Georgia, USA

17. Zewditu Memorial Hospital, Department of Surgery, Addis Ababa, Ethiopia

## 18. Hospital San José de Osorno, Department of Anesthesia, Los Lagos, Chile

Lifebox Clean Cut Program Management Collaborators: Milena Abreha, Constanza Aguilera, Bella Lima, Hillena Kebede, MD MPH

**Supplementary Figures and Tables**

**Supplementary Table 1.** Case mix among patients meeting inclusion criteria

| Case mix (N=8714) | | |
| --- | --- | --- |
| **General surgery** | 628 | 7.2% |
| Breast | 34 | 0.4% |
| Colorectal surgery | 199 | 2.3% |
| Foregut and hepatobiliary surgery | 109 | 1.3% |
| Hernia | 194 | 2.2% |
| Skin or soft tissue surgery | 85 | 1.0% |
| Trauma | 7 | 0.1% |
|  |  |  |
| **Subspecialty surgery** | 501 | 5.7% |
| Head and neck surgery | 171 | 2.0% |
| Thoracic surgery | 2 | 0.0% |
| Urologic | 56 | 0.6% |
| Neurosurgery | 79 | 0.9% |
| Orthopedic surgery | 123 | 1.4% |
| Vascular surgery | 70 | 0.8% |
|  |  |  |
| **Gynecologic surgery** | 697 | 8.0% |
|  |  |  |
| **Obstetric surgery (cesarean section)** | 6,888 | 79.0% |

**Supplementary Table 2.** Patient characteristics among patients lost to follow-up and among those with complete follow-up

|  | **Lost to follow-up** | **Followed-up** |
| --- | --- | --- |
| N | 3889 | 4825 |
| Recorded Age DHIS2, mean (SD) | 28.6 (9.2) | 27.8 (12.1) |
| Sex |  |  |
| Female | 3761 (96.7%) | 4276 (88.6%) |
| Male | 128 (3.3%) | 551 (11.4%) |
| Hypertension | 145 (3.7%) | 205 (4.2%) |
| Diabetes | 30 (0.8%) | 74 (1.5%) |
| Wound class |  |  |
| 1 | 1823 (46.9%) | 2490 (51.6%) |
| 2 | 2066 (53.1%) | 2337 (48.4%) |
| ASA Classification |  |  |
| I | 2064 (53.2%) | 3149 (66.7%) |
| II | 1757 (45.3%) | 1447 (30.7%) |
| III | 55 (1.4%) | 122 (2.6%) |
| IV | 1 (<1%) | 1 (<1%) |
| Procedure group |  |  |
| General Surgery | 159 (4.1%) | 469 (9.7%) |
| Obstetric | 3283 (84.4%) | 3607 (74.7%) |
| Subspecialty Surgery | 90 (2.3%) | 411 (8.5%) |
| Gynecology | 357 (9.2%) | 340 (7.0%) |
| Type of case: elective vs. Emergent |  |  |
| Elective | 1094 (28.1%) | 1758 (36.4%) |
| Emergency | 2795 (71.9%) | 3069 (63.6%) |
| Mean Compliance Score | 3.3 (1.5) | 3.0 (1.5) |

**Supplementary Table 3.** Results of best- and worst-case scenario sensitivity analyses. All patients lost to follow-up were assumed to have an SSI in the worst-case, while all patients were assumed not to have an SSI in the best-case.

|  | Worst case scenario: assuming all those lost to follow-up were "YES" for SSI (N=8714) | | | Worst case scenario: assuming all those lost to follow-up were "NO" for SSI (N=8714) | | |
| --- | --- | --- | --- | --- | --- | --- |
|  | RR | 95% CI | p-value | RR | 95% CI | p-value |
| **Length of postoperative antibiotic prophylaxis** |  |  |  |  |  |  |
| Less than 24 hours (ref) | 1 |  |  | 1 |  |  |
| More than 24 hours | 0.95 | (0.887 - 1.023) | 0.178 | 1.17 | (0.963 - 1.430) | 0.114 |
|  |  |  |  |  |  |  |
| **Sex** |  |  |  |  |  |  |
| Female | 0 |  |  | 0 |  |  |
| Male | 0.87 | (0.714 - 1.071) | 0.195 | 1.27 | (0.881 - 1.820) | 0.202 |
|  |  |  |  |  |  |  |
| **Age (years)** |  |  |  |  |  |  |
| Less than 20 (ref) | 1 |  |  | 1 |  |  |
| 20 - 29 | 1.35 | (1.173 - 1.558) | <0.001 | 1.37 | (0.962 - 1.956) | 0.081 |
| 30 - 39 | 1.34 | (1.158 - 1.552) | <0.001 | 1.38 | (0.961 - 1.995) | 0.081 |
| 40 or older | 1.46 | (1.225 - 1.733) | <0.001 | 1.20 | (0.804 - 1.800) | 0.369 |
|  |  |  |  |  |  |  |
| **Hypertension** | 0.97 | (0.833 - 1.131) | 0.699 | 1.22 | (0.825 - 1.796) | 0.321 |
|  |  |  |  |  |  |  |
| **Diabetes** | 0.91 | (0.675 - 1.228) | 0.539 | 1.83 | (1.062 - 3.162) | 0.030 |
|  |  |  |  |  |  |  |
| **Procedure type** |  |  |  |  |  |  |
| General surgery (ref) | 1 |  |  | 1 |  |  |
| Obstetric | 1.18 | (0.973 - 1.430) | 0.093 | 0.66 | (0.434 - 1.000) | 0.050 |
| Subspecialty Surgery | 0.95 | (0.773 - 1.168) | 0.625 | 2.12 | (1.463 - 3.079) | 0.000 |
| Gynecology | 1.29 | (1.043 - 1.585) | 0.019 | 0.77 | (0.459 - 1.291) | 0.322 |
|  |  |  |  |  |  |  |
| **Adherence with perioperative infection prevention** |  |  |  |  |  |  |
| Low adherence (<3 of 6 standards met) (ref) | 1 |  |  | 1 |  |  |
| High adherence (3 or more standards met) | 1.10 | (1.033 - 1.179) | 0.004 | 0.67 | (0.557 - 0.807) | 0.000 |
|  |  |  |  |  |  |  |
| **Wound class** |  |  |  |  |  |  |
| Clean (ref) | 0 |  |  | 0 |  |  |
| Clean contaminated | 1.08 | (1.012 - 1.145) | 0.019 | 1.17 | (0.976 - 1.398) | 0.091 |
|  |  |  |  |  |  |  |
| **Case urgency** |  |  |  |  |  |  |
| Elective |  |  |  |  |  |  |
| Urgent or emergent | 1.16 | (1.073 - 1.248) | <0.001 | 1.29 | (1.029 - 1.605) | 0.027 |

**Supplementary Table 4.** Indication for antibiotics prescribed among patients who received antibiotics for reasons other than prophylaxis

| **Indication for antibiotics** | **N = 99** | **Percent of total patients** |
| --- | --- | --- |
| Chorioamnionitis | 17 | 17% |
| Endometritis | 5 | 5% |
| Meningitis | 1 | 1% |
| Pneumonia | 8 | 8% |
| Surgical site infection | 59 | 60% |
| UTI | 9 | 9% |
